# Vital Statistics of Kentucky

**Published:** 1854-04

**Authors:** 


### Art. V.—VITAL STATISTICS OF KENTUCKY.

We have before us the "First Annual Report relating to the Registry and Returns of the Births, Marriages and Deaths, in Kentucky, in 1852," and we have examined its tables with great interest. The profession is indebted for the report to Dr. Sutton, of Georgetown, whose unwearied devotion to this subject entitles him to the thanks of every friend of medical science. The State Auditor, Thomas S. Page, Esq., the duties of whose office, it has been computed, would occupy a less efficient man twenty-three hours and forty-five minutes every day, finding that it would be impossible for him to digest and arrange the Assessor's returns in the time prescribed by law, very wisely determined to procure the services of Dr. Sutton, under whose supervision and direction the report has been prepared. Whatever imperfections may be found in the report are chargeable to the Assessors, some of whom were far from being careful in making out their returns, and among these, we are sorry to say, is the officer who prepared the schedule for the populous county of Jefferson. But the system is new in Kentucky, and it was not to be expected that it would work perfectly at the start, both physicians and public officers requiring time to become familiar with their respective duties. We have said that the deficiencies in this report are due to the Assessors, but we are not sure that we should not have been nearer the truth if we had taken the chief blame to the members of our profession. As it is, Dr. Sutton believes "the returns are more full than those produced by any State heretofore at the commencement," and we are quite confident they will be more so in future.

The population of Kentucky in 1850 was very nearly a million; in 1852 the births amounted to 25,907, the deaths to 13,848, being an excess of births over deaths of 12,058. The mortality as distributed over the several months of the year was least in February, 643, and at its height in August, when it reached 1805. It then declined through the following months. As to age, 4,982, or more than a third, died before reaching five years; between 5 and 10 years only 1,014 died, and between 10 and 15 only 700, after which the mortality began to mount up, being for the next five years, or among those from 15 to 20, over 800, and for the next ten years, 20 to 30, nearly 1500. Of persons from 40 to 50, 650 died, from 50 to 60, 551, from 60 to 70, 485, from 70 to 80, 418, over 80, 310. Twelve are reported as having passed 100, and one, a white female, as having attained 110 years.

In regard to the births, Dr. Sutton says:—

"Of these 25,734 whose sex is stated, 13,625 were males, 12,-109 females—giving the very remarkable proportion of 112.52 males to 100 females. If there is any instance upon record of so large a proportion of males in a population of a million, I do not know where it is. In every month in the year there was a majority of males. But in June this majority was only 3 in 1,891 children born, whilst in December it was 209 in 2,691 births, or 116.84 to 100.

"The excess of male births is very remarkable in both the plurality cases and in the still-born. Of 230 twins whose sex is stated, 142 are males, 88 females, or 161,36 males to 100 females. Of 785 still-born, whose sex is stated, 481 are males and 304 females, or 158.22 males to 100 females.

"No case of triplets is recorded in the whole State. One woman (colored) is reported as having borne a child at 11 years.

"The still-born certainly appear to be in large proportion—no less than 3.09 per cent. of all births. This may be so; yet there is reason to believe that it is too large, beeause a number of children are returned as still-born who have names. There is reason to believe that a number of assessors mistook the precise import of the terms 'alive' and 'dead,' and returned as dead those which were dead at the time of making the assessment."

Respecting the relative mortality of different counties he remarks:—

"We find the mortality much greater in some portions of the State than in others. Muhlenburg, Union, and Jefferson report the smallest mortality, viz: one in 817, 391, and 291, respectively. It mast be observed, however, that the schedules from each of these counties bear very strong evidence of having been very negligently taken. Hickman, Bourbon, Hart, Oldham, and Marshall, have returned the largest mortality, varying from one death in 34.22 to one in 43.54. As might reasonably have been expected, counties have changed their position from that which they occupied in the census of 1850. Fayette, which then exhibited a mortality much larger than any other county in the State, now exhibits a mortality less than one per cent.; and Hickman, which then was less than one per cent., has now the greatest mortality, nearly three per cent."

*The Causes of Death.*—We quote Dr. SUTTON's recapitulation under this head:—

"In examining table X, we shall be forcibly struck by the fatality occasioned by class 1, ZYMOTICS. This gives a mortality of 56.43 per cent. of all ascertained causes of death. This class is large in all countries, but very rarely assumes the magnitude here displayed. In Massachusetts it has ranged from 27 to 38 per cent.; the average for 10 years being 30.42 per cent. This class contains diseases which it is thought can be very much guarded against by proper sanitary regulations, and therefore ought not to be permitted to produce such an awful mortality.

"At the head of the alphabetical list in this class we have *Cholera*, which is represented by a mortality of 722, or 6.94 per cent. By running the eye along the list of counties, it will be seen that this disease prevailed to any considerable effect in only a few counties. A few cases of cholera morbus are included under this head.

"Next comes *Cholera Infantum*, which twenty or thirty years ago was a great outlet of infant life. Of late years, the mortality from this cause has very much diminished; but 135 cases, or 1.30 per cent. of deaths, is ascribed to it.

"*Croup* occupies a more prominent place, having caused 461 deaths, or 4.53 per cent. of deaths.

"*Diarrhœa* occupies but little space; but *Dysentery* stands forth in bold relief—no less than 1,923, or 18.47* per cent. having died of it.

"Of *Fever*, intermittent, remittent, and continued fever, the last of which contains typhus and typhoid fevers, we may speak in a group—continued fever having produced a greater mortality than any disease, except dysentery; and if we include (what probably enough belong there) all entries under 'fever' simply, we have no less than 13.09 per cent. of deaths from all ascertained causes. Remittent fever, which includes all entries under the heads of bilious fever, congestive fever, and one of yellow fever, gives a mortality of 2.07 per cent.

"The fevers above mentioned, with cholera, cholera infantum, diarrhœa, and dysentery, constitutes the great outlet of life—affording as they do 43.27 per cent. of all deaths. It is generally admitted that these diseases are dependent upon the same causes, more or less modified; and further, that these causes are very considerably under the control of man. Why then should they not be looked to and obviated?"

*The per cent. of each disease was calculated before the returns from Letcher county came in. This slightly increased the per cent. of dysentery, but did not sensibly affect any other disease.

